# Changes in microbiome diversity following beta-lactam antibiotic treatment are associated with therapeutic versus subtherapeutic antibiotic exposure in cystic fibrosis

**DOI:** 10.1038/s41598-019-38984-y

**Published:** 2019-02-22

**Authors:** Andrea Hahn, Hani Fanous, Caroline Jensen, Hollis Chaney, Iman Sami, Geovanny F. Perez, Anastassios C. Koumbourlis, Stan Louie, James E. Bost, John N. van den Anker, Robert J. Freishtat, Edith T. Zemanick, Keith A. Crandall

**Affiliations:** 1 Division of Infectious Diseases, Children’s National Health System (CNHS), 111 Michigan Ave NW, Washington DC, 20010 USA; 2 Department of Pediatrics, George Washington University (GWU) School of Medicine and Health Sciences, 2300 Eye Street NW, Washington DC, 20037 USA; 3 Division of Pulmonary and Sleep Medicine, CNHS, 111 Michigan Ave NW, Washington DC, 20010 USA; 4 GWU School of Medicine and Health Sciences, 2300 Eye Street NW, Washington DC, 20037 USA; 5 Department of Clinical Pharmacy, University of Southern California School of Pharmacy, 1985 Zonal Ave, Los Angeles, CA 90089 USA; 6 Division of Biostatistics and Study Methodology, CNHS, 111 Michigan Ave NW, Washington DC, 20010 USA; 7 Division of Clinical Pharmacology, CNHS, 111 Michigan Ave NW, Washington DC, 20010 USA; 8 Division of Emergency Medicine, CNHS, 111 Michigan Ave NW, Washington DC, 20010 USA; 9 Department of Pediatrics, University of Colorado Anschutz Medical Campus, 13123 E. 16th Ave, Aurora, CO 80045 USA; 10 Computational Biology Institute, GWU, 45085 University Drive, Ashburn, VA 20147 USA

## Abstract

In persons with cystic fibrosis (CF), decreased airway microbial diversity is associated with lower lung function. Conflicting data exist on the impact of short-term antibiotics for treatment of acute pulmonary exacerbations. However, whether differences in antibiotic exposure impacts airway microbiome changes has not been studied. We hypothesized that subtherapeutic beta-lactam antibiotic exposure, determined by the pharmacokinetics and pharmacodynamics (PK/PD) after intravenous (IV) antibiotic administration, would be associated with different patterns of changes in CF airway microbial diversity. Eligible children were enrolled when well; study assessments were performed around the time of pulmonary exacerbation. Plasma drug concentrations and bacterial minimum inhibitory concentrations (MICs) were used to determine therapeutic versus subtherapeutic beta-lactam antibiotic exposure. Respiratory samples were collected from children, and extracted bacterial DNA was amplified for the V4 region of the 16S rRNA gene. Twenty children experienced 31 APEs during the study; 45% (n = 14) of antibiotic courses were deemed therapeutic. Those in the therapeutic group had more significant decreases in alpha diversity at end of treatment and post-recovery compared to baseline than those in the subtherapeutic group. Therapeutic and subtherapeutic beta-lactam use is associated with different patterns of changes in CF airway microbial diversity following antibiotic administration.

## Introduction

More than 30,000 people in the United States are living with cystic fibrosis (CF), a severe autosomal recessive disease that leads to recurrent lung infections and chronic suppurative lung disease associated with significant morbidity and mortality^1,2^. Children and adults frequently require hospitalization for severe recurrent lung infections, known as acute pulmonary exacerbations (APE)^3,4^. Antibiotic use is typically directed at specific pathogens, such as *Pseudomonas aeruginosa*^5^. However, prior studies have shown that *in vitro* susceptibilities do not correlate with clinical outcomes^6^.

This inconsistency may be due in part to the numerous other bacteria such as *Prevotella spp*., *Veillonella spp*., and *Gemella spp*. that have commonly been identified in CF airways by culture-independent sequencing^7–13^. These obligate and facultative anaerobic bacteria may be positively associated with the development of APE^7^. The impact of antibiotics on microbial diversity and dominant core bacteria within the airway is unclear. Some studies suggest minimal changes within the bacterial community overall and resilience of core bacteria^8,14,15^, while others show decreases in *Pseudomonas spp*. when present^16,17^.

The variations in treatment response and airway microbiome changes to intravenous (IV) antibiotic therapy also may be due in part to subtherapeutic antibiotic exposure. The most common IV antibiotics used for treatment of APE in CF is a combination of an anti-pseudomonal beta-lactam antibiotic and an aminoglycoside^5,18,19^. Bacterial killing by beta-lactam antibiotics is time-dependent, meaning that the serum concentration of the antibiotic must be above the minimum inhibitory concentration (MIC) of the bacteria for a certain period of time (T > MIC)^20–24^. Due to higher beta-lactam antibiotic clearance in CF persons, the CF Foundation (CFF) and the European consensus guidelines recommend higher dosing regimens to achieve the needed serum concentrations^25,26^. However, surveys of CF centers in the US found that IV anti-pseudomonal beta-lactams were dosed below these guidelines 38–53% of the time^18,27^. No studies have evaluated IV beta-lactam antibiotic pharmacokinetics (PK) and pharmacodynamics (PD), specifically T > MIC, on relative bacterial abundance and alpha diversity.

Identifying risk factors for decreasing microbial diversity in the CF airway is important because decreased alpha diversity is clearly associated with disease progression^8,28–30^. However, the impact of antibiotics for acute treatment of APE on microbial diversity is less clear, with prior studies showing conflicting results during antibiotic treatment^8,14–17^. Furthermore, the impact of temporary decreases in microbial diversity with short courses of antibiotics with rebound to pre-treatment levels is not known. We hypothesized that subtherapeutic IV beta-lactam antibiotic exposure determined by PK/PD, and defined as insufficient time above the minimum inhibitory concentration (MIC) of the bacteria grown in culture, would be associated with different patterns of changes in CF airway microbial diversity. Specifically, we expected that subtherapeutic beta-lactam antibiotic exposure would have less of an impact on bacterial communities and diversity due to a lack of change in relative abundance of the targeted bacteria compared to therapeutic exposure.

## Results

### Determination of therapeutic versus subtherapeutic beta-lactam antibiotic exposure

Twenty study participants age 1 to 21 years of age were treated with 31 courses of IV antibiotics during the study period. The majority of participants (n = 13) had only one APE, but 5 participants had 2 APEs, 1 participant had 3 APEs, and 1 participant had 5 APEs.

Of the 31 antibiotic regimens administered, 25 included a single beta-lactam antibiotic. Five courses included coverage with two beta-lactams, while one course included 3 beta-lactams for more than 72 hrs. All beta-lactams were intermittently dosed and none were given via extended or continuous infusion. Time above MIC (T > MIC) was determined for all beta-lactams; those patients with at least one beta-lactam with appropriate PD indices were considered therapeutic. Of the 5 patients who grew MRSA, three were on appropriate MRSA-directed therapy. The other two patients were already categorized as subtherapeutic based on their beta-lactam T > MIC indices and did not have to be re-categorized. Of note, 81% of all study participants also received an aminoglycoside as part of their antibiotic regimen that had been adjusted per our institutional guideline to meet goal peak concentrations. As aminoglycoside use was not different between the therapeutic and subtherapeutic groups (79 vs 82%, p > 0.999), it was not considered in our categorization of therapeutic versus subtherapeutic.

Ultimately, 45% (n = 14) of the antibiotic treatment regimens achieved optimal PD indices and were considered therapeutic, while 55% (n = 17) did not.

### 16S rRNA sequencing of the CF airway microbiome

Respiratory samples [sputum, oropharyngeal (OP) swabs or bronchoalveolar lavage fluid (BAL)] were collected for 16S rRNA sequencing. Across all samples collected, 35% (n = 18) in the therapeutic group were sputum and 65% (n = 33) were OP swabs. In the subtherapeutic group 79% (n = 41) of the samples were sputum, 17% (n = 9) were OP swabs, and 4% (n = 2) were from BAL fluid. A sensitivity analysis of sample type on diversity measures was performed on samples from 4 participants with discordant sample types collected (see Supplemental Results). Based on these findings, we excluded one OP swab and one sputum sample from further analysis. Lastly, for eleven time points, respiratory samples were not collected (n = 10) or failed sequencing (n = 1) and were also excluded from subsequent analysis.

### Baseline participant characteristics

There was no significant difference in age, gender, race/ethnicity, or CFTR genotype between therapeutic and subtherapeutic participants (Table 1). Those in the therapeutic group were significantly less likely to be receiving inhaled antibiotics at baseline compared to those in the subtherapeutic group (45% vs 100%, p = 0.01). Those in the therapeutic group had higher baseline percent predicted forced expiratory volume in one second (FEV_1_) and forced vital capacity (FVC) compared to those in the subtherapeutic group (FEV_1_ 90.1 vs 65.1%, p = 0.02 and FVC 98.3 vs 74.2%, p = 0.01).

**Table 1:** Study participant characteristics at baseline.

	Therapeutic* (N = 11)	Subtherapeutic (N = 9)	P-value
Sex (n,% female)	3 (27%)	3 (33%)	0.769
Age (median, range in years)	9 (1–19)	14 (6–21)	0.287
Race /Ethnicity (n, %)			0.178
White	7 (63%)	3 (33%)	
Non-white^#^	4 (36%)	6 (67%)	
CFTR Mutation (n, %)			0.790
F508del homozygous	3 (27%)	3 (34%)	
F508del heterozygous	4 (36%)	4 (44%)	
Other	4 (36%)	2 (22%)	
On suppressive inhaled antibiotics (n, %)			0.014
Yes	5 (45%)	9 (100%)	
No	6 (55%)	0 (0%)	
Pulmonary Function, % predicted (mean, SD)^†^			
FEV_1_	90.1 (17.3)	65.1 (22.4)	0.022
FVC	98.3 (17.1)	74.2 (17.0)	0.010
FEV_1_/FVC	81.1 (8.1)	76.9 (13.8)	0.465
FEF_25–75_	75.7 (23.2)	56.3 (35.1)	0.207
Respiratory Sample Type (n, % sputum)	4 (36%)	8 (89%)	0.028
Alpha Diversity (mean, SD)^||^			
No. genus	31.6 (11.1)	22.1 (11.5)	0.076
Ace	38.1 (16.5)	25.3 (12.4)	0.072
Chao	35.4 (14.4)	24.7 (12.3)	0.097
Shannon Diversity Index	2.189 (0.191)	1.551 (0.313)	0.087
Inverse Simpson Index	6.294 (1.089)	4.565 (1.532)	0.358

*Therapeutic and subtherapeutic categorization was based on each participants’ first acute pulmonary exacerbation (note of the 20 participants, for those with multiple exacerbations, only two had different categorizations).

^#^Non-white included Hispanic (4/4 therapeutic, 5/6 subtherapeutic) and African-American (1/6 subtherapeutic).

^†^Only 10 of 11 patients in the therapeutic group had pulmonary function tests obtained. FEV_1_, forced expiratory volume in one second; FVC, forced vital capcity; FEF_25–75_, forced expiratory flow at 25–75%.

^||^No. genus (number of genera detected), Ace (abundance coverage estimator), and Chao are all measures of richness (q = 0). Shannon Diversity Index equally weights richness and evenness (q = 1). Inverse Simpson Index provides more weight to evenness (q = 2).

**Figure 1:**
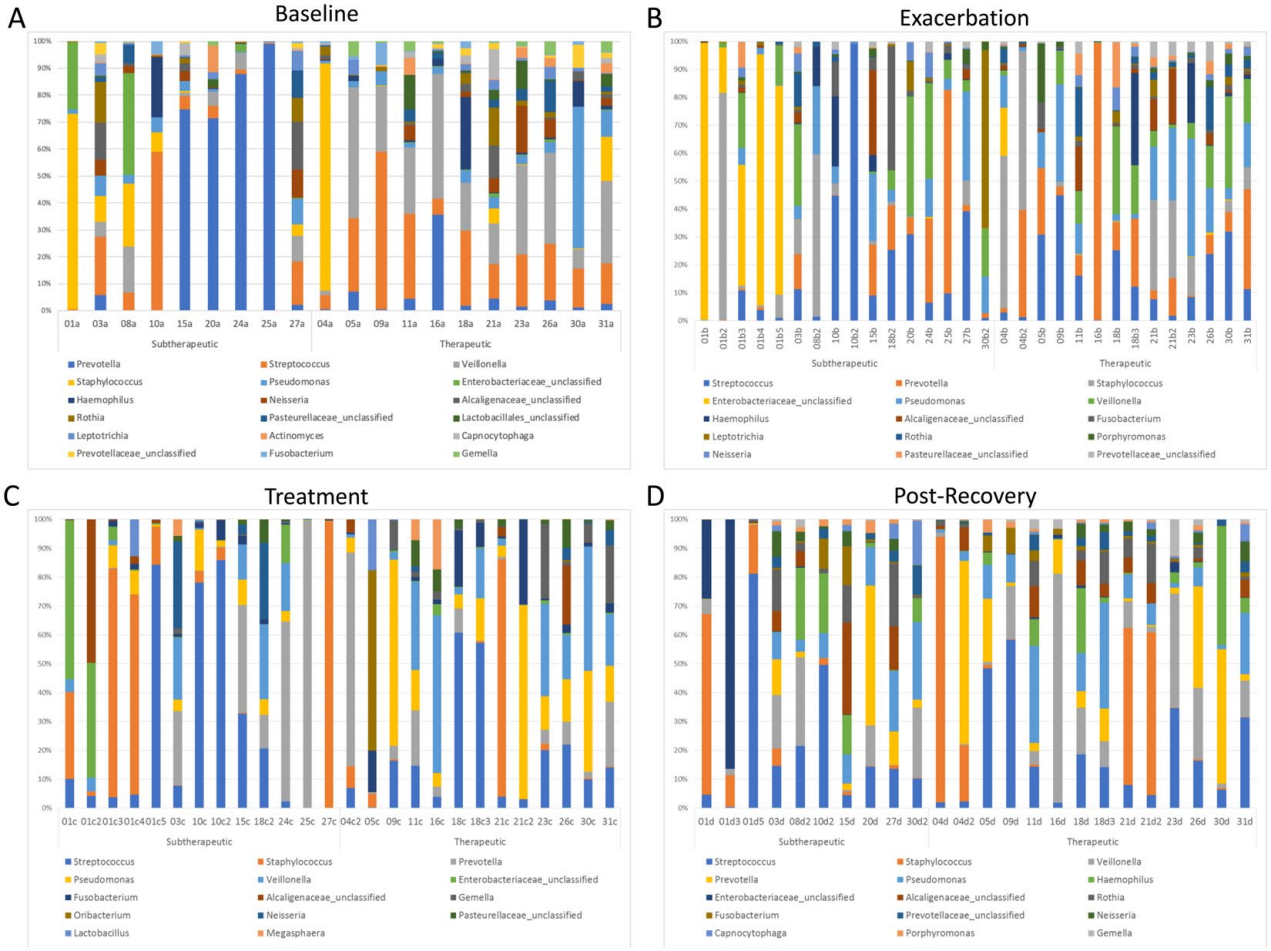
Relative abundance of bacterial genera. Panel A. Baseline. Panel B. Exacerbation. Panel C. Treatment. Panel D. Post-Recovery. Only bacterial genera that contributed to >1% of the total sequencing reads are included.

The relative abundance of bacterial genera identified by sequencing in the baseline samples is shown in Fig. 1A. Those in the therapeutic group also had significantly lower relative abundance of the operational taxonomic unit (OTU) defined as the genera *Enterobacteriaceae*_unclassified compared to the therapeutic group (log2 fold difference = −5.31, adjusted p = 0.002). There was also a trend that those with therapeutic group had higher alpha diversity measures at baseline compared to those in the subtherapeutic group (Table 1).

### Exacerbation characteristics

There was no significant difference in the presence of *Pseudomonas aeruginosa* or *Staphylococcus aureus* in respiratory culture results at exacerbation onset between the therapeutic and subtherapeutic groups (Table 2). There was a trend toward normal flora being higher in the respiratory cultures of the therapeutic group across exacerbations (43% vs 12%, p = 0.09), and other pathogens being present more frequently in the respiratory cultures of the subtherapeutic group (59% vs 21%, p = 0.09) (Table 2, Supplemental Fig. 1). The relative abundance of bacterial genera determined by sequencing are shown in Fig. 1B. There was a significant increase in the relative abundance of *Gemella* and a decrease of Enterobacteriaceae_unclassified in the therapeutic group compared to the subtherapeutic group (log 2 fold change 3.40, adjusted p = 0.03, and log 2 fold change −6.53, adjusted p < 0.001, respectively). Alpha diversity was also significantly higher in the exacerbation samples of those in the therapeutic group compared to the subtherapeutic group (Chao 38 vs 24.9, p = 0.02, Shannon 2.11 vs 14.8, p = 0.05, and Inverse Simpson 6.24 vs 3.81, p = 0.04.

**Table 2:** Cultured pathogens, alpha diversity, and beta-lactam antibiotic use during treatment of acute pulmonary exacerbation.

	Therapeutic* (N = 14)	Subtherapeutic (N = 17)	P-value
Cultured Pathogen (n, %)			
*Pseudomonas aeruginosa*	3 (21%)	5 (29%)	0.318
*Staphylococcus aureus*	5 (36%)	6 (35%)	0.940
Other Pathogen^#^	3 (21%)	10 (59%)	0.091
Normal Flora^†^	6 (43%)	2 (12%)	0.087
Alpha diversity (mean, SD)^§^			
No. Genus	32.6 (13.5)	19.8 (11.7)	0.091
Ace	39.8 (15.1)	25.5 (15.4)	0.073
Chao	38.0 (15.6)	24.9 (16.8)	0.020
Shannon	2.11 (0.79)	1.48 (0.86)	0.048
Inverse Simpson	6.24 (3.19)	3.81 (2.60)	0.037
Beta-lactam antibiotics used (n, %)			
Ceftazidime	12 (86%)	7 (41%)	0.053
Meropenem	3 (21%)	10 (59%)	0.052
Piperacillin-tazobactam	1(7%)	5 (29%)	0.189
Antibiotic Days (mean, SD)	13.7 (7.7)	18.8 (6.4)	0.107
Days from baseline/post-recovery to exacerbation (mean, SD)^ǂ^	135.3 (75.3)	135.5 (115.5)	0.583
Days from end of treatment to post-recovery (mean, SD)^ǂ^	50.6 (25.8)	79.3 (56.5)	0.003

*Therapeutic and subtherapeutic categorization was based on each acute pulmonary exacerbation.

^#^See Supplemental Fig. 1.

^†^For patients whose cultures only grew normal flora, the median MIC for Pseudomonas aeruginosa in the cohort was used to determine T > MIC.

^§^Alpha diversity measures were calculated with N = 11 in the therapeutic group and N = 16 in the subtherapeutic group. No. genus (number of genera detected), Ace (abundance coverage estimator), and Chao are all measures of richness (q = 0). Shannon Diversity Index equally weights richness and evenness (q = 1). Inverse Simpson Index provides more weight to evenness (q = 2).

^ǂ^Missing data points occurred when a patient’s follow up visit was also a new exacerbation.

While there were no statistically significant differences in the beta-lactam antibiotics administered between the two groups, ceftazidime (a more narrow spectrum beta-lactam) was used more often in the therapeutic group (86 vs 41%, p = 0.05), while meropenem (a broad spectrum beta-lactam with substantial anaerobic coverage) was used less often (21 vs 59%, p = 0.05) (Table 2). There were no significant differences in total antibiotic days or the days between collection of the baseline samples to the onset of exacerbation requiring IV antibiotic therapy between the therapeutic and subtherapeutic groups (Table 2). Those in the therapeutic group had a significantly shorter time from end of treatment to post-recovery compared to those in the subtherapeutic group (51 vs 79 days, p = 0.003).

### Changes in alpha diversity and community composition following antibiotic administration (Treatment and Post-recovery) relative to Baseline samples

**Figure 2:**
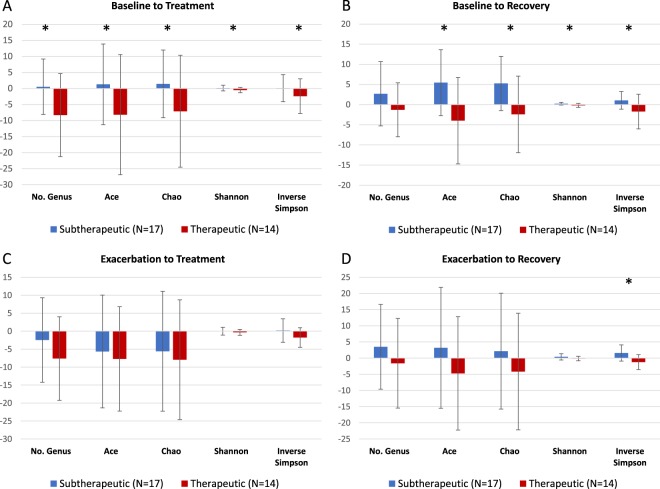
Modeling the impact of therapeutic versus subtherapeutic treatments on airway alpha diversity measures. Panel A. Baseline to Treatment. Panel B. Baseline to Recovery. Panel C. Exacerbation to Treatment. Panel D. Exacerbation to Recovery. The bar graph represents the mean alpha diversity measure, and the error bars represent the standard deviation. *P < 0.05.

We sought to identify differences in response to antibiotic therapy based on therapeutic versus subtherapeutic antibiotic exposure. Changes in alpha diversity between baseline and treatment samples and baseline and post-recovery samples are shown in Fig. 2. Significant differences were found for all measurements between the baseline and treatment samples, with those in the therapeutic group having a decrease in diversity at treatment compared to baseline, compared to those in the subtherapeutic group who had a slight increase or minimal change in diversity (Fig. 2A, Supplemental Table 1). Similar significance was found when comparing baseline to post-recovery samples; those in the therapeutic group had slight decreases in overall diversity but those in the subtherapeutic group had increases in diversity (Fig. 2B, Supplemental Table 1).

We also evaluated for significant changes (adjusted p-value < 0.05) in the abundance of bacterial genera between baseline and treatment and baseline and post-recovery for the therapeutic and subtherapeutic groups separately. Those in the therapeutic group had a higher relative abundance of the following bacterial genera in baseline versus treatment samples: *Haemophilus* (log 2 fold change 3.63, p = 0.004), Clostridiales_unclassified (log 2 fold change 3.12, p = 0.010), and Lachnospiraceae_unclassified (log 2 fold change 2.37, p = 0.033). Interestingly, a lower relative abundance of *Fusobacterium* (log 2 fold change −3.08, p = 0.001) and *Pseudomonas* (log 2 fold change −2.30, p = 0.021) were found in the baseline samples compared to treatment samples. No significant differences were found between baseline and treatment samples for those in the subtherapeutic group. There were also no significant differences identified between baseline and post-recovery samples for either group.

**Figure 3:**
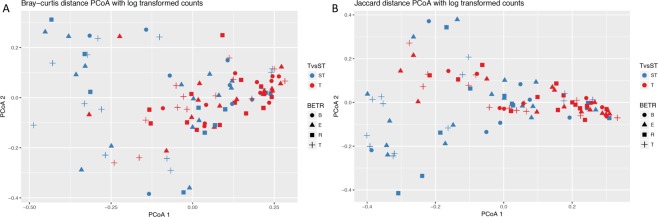
Bray-curtis and Jaccard distance PCoA with log transformed counts. Dissimilarities in bacterial genera are shown based on antibiotic exposure and timing of sample collection. Panel A. Bray-curtis distance PCoA for therapeutic (T) versus subtherapeutic (ST) antibiotic exposure (p = 0.003) and baseline (B), exacerbation (E), treatment (T), and recovery (R) time of sample collection (p = 0.722). Panel B. Jaccard distance PCoA for therapeutic (T) versus subtherapeutic (ST) antibiotic exposure (p = 0.003) and baseline (B), exacerbation (E), treatment (T), and recovery (R) time of sample collection (p = 0.627). P-value was determined using the *adonis* function of vegan in R.

Analysis of beta diversity using Morisita-Horn showed no significant differences between those in the therapeutic and subtherapeutic groups (Supplemental Fig. 2). Dissimilarities using log-transformed Bray-Curtis distance (Fig. 3A) and Jaccard distance (Fig. 3B) between all samples collected for the therapeutic and subtherapeutic groups are shown in a principal coordinates analysis (PCoA). There was a significant difference between the therapeutic and subtherapeutic groups determined by a permutational multivariate analysis variance using distance matrices (Bray distance p = 0.003, Jaccard distance p = 0.003). This significance between the therapeutic and subtherapeutic groups also held true for baseline samples alone (Bray distance p = 0.01, Jaccard distance p = 0.005), and almost reached significance for treatment samples (Bray distance p = 0.07, Jaccard distance p = 0.09).

### Changes in alpha diversity and community composition following antibiotic administration (Treatment and Post-Recovery) relative to Exacerbation samples

We next sought to identify differences between the therapeutic and subtherapeutic groups when comparing changes in alpha diversity between exacerbation and treatment samples and exacerbation and post-recovery samples (Fig. 2, Supplemental Table 2). No significant differences were noted for all measurements between the exacerbation and treatment samples (Fig. 2C, Supplemental Table 2), with both groups experiencing a decrease in diversity. When comparing changes in alpha diversity between exacerbation and post-recovery samples, those in the therapeutic group tended to have decreases in diversity while those in the subtherapeutic group had increases (Fig. 2D, Supplemental Table 2). However, only the Inverse Simpson index was statistically significant (−1.24 vs 1.59, p = 0.003). Analysis of beta diversity using Morisita-Horn also showed no significant differences between those in the therapeutic and subtherapeutic groups (Supplemental Fig. 2).

When we evaluated for changes in the abundance of bacterial genera between exacerbation and treatment samples, we found no significant differences for either the therapeutic or subtherapeutic groups. This was also true when we evaluated for changes between exacerbation and post-recovery samples.

### Changes in pulmonary function following antibiotic administration

Lastly, we analyzed the impact of therapeutic versus subtherapeutic beta-lactam exposure on changes in normalized pulmonary function (Supplemental Fig. 3). While no statistical differences were noted, trends were present that showed patients receiving therapeutic antibiotics had a higher relative improvement of FEV_1_, and FEV_1_/FVC at post-recovery compared to onset of the exacerbation than those in the subtherapeutic group (FEV_1_ 28.8 vs 14.8, p = 0.11; FEV_1_/FVC 9 vs 1.1, p = 0.07). This corroborates our findings previously published on this same cohort of patients where significant differences were found in non-normalized pulmonary function between the two groups, with higher recovery in those achieving therapeutic beta-lactam drug exposure^31^.

## Discussion

We found that children with CF who achieved subtherapeutic beta-lactam antibiotic exposure had minimal change in microbial diversity compared to baseline samples during the IV antibiotic treatment course and higher airway microbial diversity more than one month after the completion of antibiotic treatment compared to their baseline samples. This is significantly different than observed in those with therapeutic antibiotic exposure, who had decreases in their microbial diversity during the IV antibiotic treatment course compared to baseline and a continued decrease in diversity levels more than one month after the completion of antibiotic treatment. This suggests that those achieving therapeutic antibiotic exposure follow an expected progression of changes in diversity in response to antibiotic exposure, with a decrease in diversity occurring during antibiotic treatment and a recovery of that diversity once antibiotics have been discontinued. This expected pattern does not seem to hold true for those achieving subtherapeutic antibiotic exposure. This may be related in part to the trend toward lower baseline diversity levels and the significantly lower diversity at exacerbation onset of those in the subtherapeutic group compared to the therapeutic group. This could represent a basement effect where it is harder to decrease diversity when it is already low to start. In addition, those in the subtherapeutic group had more advanced disease than those in the therapeutic group, as signified by lower pulmonary function. These baseline differences between our two groups may account for some of the differences we found in our diversity and outcome measures. However, these findings more clearly establish the importance of baseline diversity and lung function when trying to interpret the impact of antibiotics for treatment of acute pulmonary exacerbations.

Prior studies of the impact of antibiotic use for treatment of APE on airway microbiome have varied in their findings. One study found that the community structure within the CF microbiome did not significantly change in response antibiotic use for APE^8^, while another found that the relative abundance of bacterial genera remained stable despite antibiotic use^14^. A third study also showed resilience of core bacteria, including *P. aeruginosa*, across the BETR spectrum^15^. However, another study found specifically in the treatment of *Pseudomonas*-dominated samples, the *Pseudomonas spp*. decreased but anaerobic genera remained stable^16^. The presence of *Pseudomonas spp*. was also thought to contribute to the finding of a short term decrease in alpha diversity (day 3 of antibiotics), followed by an increase in diversity by day 8–10 of antibiotic therapy in another study^17^.

Several studies have shown that decreased microbial diversity is associated with more severe lung disease^8,28–30^. One study of longitudinal samples obtained in children and adults found that older adults with decreased lung function had more uneven bacterial abundance, measured by Pielou’s evenness, and that alpha diversity, measured as Inverse Simpson’s diversity index, decreased over time^29^. Another study found a strong correlation between poor lung function and low species richness^8^, while two others showed that lower alpha diversity, measured by Shannon diversity index, was associated with worsening pulmonary function^28,30^. Our previously published analysis of this patient cohort comparing antibiotic PK and lung function found subtherapeutic beta-lactam antibiotic exposure was associated with less improvement of lung function^31^. In this study, we also saw similar trends using normalized lung function data. Thus, the conclusion should not be drawn that because subtherapeutic antibiotics have less impact on changes in microbial diversity, it could be used as a strategy to prevent declining lung function. Further work needs to be done to determine if a strategy of using more targeted antibiotic therapy to assist in maintaining community diversity might impact CF disease progression^32^.

In the above studies, the presence of *Pseudomonas spp*. was strongly associated with decreasing diversity. In our cohort there was no significant difference noted for *Pseudomonas* between the therapeutic or subtherapeutic groups when looking at culture data obtained at the onset of exacerbation. Interestingly, in our patient population, the microbiome of those patients in the subtherapeutic group had a higher relative abundance of Enterobacteriaceae at the onset of exacerbation compared to those in the therapeutic group. Other studies have shown increased airway inflammation in the presence of Enterobacteriaceae in culture^11^, and Enterobacteriaceae have been associated with the production of beta-lactamases in CF patients^33^.

Lastly, the microbial composition of samples obtained from patients achieving therapeutic antibiotic exposure was significantly different from those achieving subtherapeutic exposure. The increased dissimilarity of microbial composition among those achieving therapeutic beta-lactam antibiotic exposure may reflect the influence of alpha diversity, as those in the therapeutic group had higher diversity at baseline and exacerbation onset yet had more significant decreases in alpha diversity at end of treatment and recovery. Surprisingly, the use of broad-spectrum beta-lactam antibiotics did not seem to play significant role in this decrease in diversity, as those in the therapeutic group were more likely to be receiving ceftazidime than meropenem.

Limitations of this study include the small study size that included a range of children with different disease severity. Those with therapeutic beta-lactam antibiotic exposure were less likely to be on inhaled antibiotics, had higher baseline diversity, and had higher lung function. These baseline differences in severity of disease may have contributed to higher bacterial MICs in the subtherapeutic group, thus making it more difficult for those with more severe disease to achieve therapeutic beta-lactam concentrations. We also found high levels of inter-patient variability, which is consistent with previously published studies. However, our analysis attempted to counter these baseline and inter-patient differences by evaluating changes of each individual study participant over time instead of raw values of microbial diversity and lung function. Future studies that evaluated beta-lactam PK/PD differences in a more homogenous cohort would help corroborate the findings of this study.

Another limitation was the use of both OP swab and sputum samples to describe the airway community. Some studies have shown that OP swab samples may underrepresent the presence of more inflammatory pathogens, such as *Pseudomonas aeruginosa*^11^. However, while in our study there were more swab samples collected in those patients achieving therapeutic antibiotic exposure, they actually had a higher relative abundance of *Pseudomonas*, suggesting our findings are unlikely to be limited by an inability to detect this bacterium. We also performed a sensitivity analysis to remove samples from analysis thought to be adversely affected by sample type. Future studies using similar respiratory sample type for all measurements would help determine the significance of using variable sample types on our study findings.

Lastly, we also relied on MIC breakpoints reported by the clinical lab, and this limited our ability to determine if higher doses of beta-lactam antibiotics could be used to overcome antibiotic resistance. This study also does not address either the variability of MICs reported within different sub-colonies of *P. aeruginosa*^34^, regional variability^35^, or antibiotic resistance within the bacterial community at large^36,37^. It is likely that one main driver behind achieving therapeutic antibiotic exposure is the bacteria’s antibiotic susceptibility as opposed to differences in antibiotic PK alone. Further studies incorporating metagenomics could provide more detailed information on bacterial drug resistant variants in the bacterial population and overcome some of the limitations of culture based methods^38,39^.

In conclusion, we found that subtherapeutic beta-lactam was associated with different patterns of changes in microbial diversity in response to antibiotic treatment. While therapeutic patients had more significant drops in alpha diversity, they trended toward improved recovery of pulmonary function. This is likely a reflection of disease severity, antibiotic exposure, and antibiotic resistance. Controlling for these factors will be important for accurately interpreting the impact of antibiotic therapy on microbial diversity and pulmonary function. Moreover, as microbial diversity is associated with disease progression, identifying additional factors that influence diversity is critical. Future studies are needed to validate the impact of antibiotic use on decreasing microbial diversity and identify ways that the CF airway microbiome might be used as a tool to better optimize antibiotic treatment for acute pulmonary exacerbations.

## Methods

### Setting and study population

This study was a prospective, longitudinal study that was conducted over 18 months at Children’s National Health System (CNHS), a tertiary care pediatric hospital serving the greater Washington, DC area. Inclusion criteria were age <21 years, having a confirmed diagnosis of CF (either genetic or positive sweat test), and having been hospitalized for IV antibiotics in the past three years (to identify those most likely to be hospitalized during the study period).

### Ethics approval and consent to participate

Institutional Board Review (IRB) at CNHS was obtained prior to beginning the study (20 Feb 2015; Pro5655). The principles outlined in the Declaration of Helsinki were followed. Written informed consent was obtained from study participants ≥18 years of age and parental permission for those <18 years of age. Assent was obtained from participants 7–17 years of age.

### Subject encounters

Study participants were evaluated at four types of research encounters. The first encounter (enrollment) represented their baseline state of health (B), and required that they had not received antibiotic treatment for an APE in the 30 days prior^12^. Inhaled maintenance antibiotics were not considered exclusionary. The second encounter began at a clinic visit or hospital admission when IV antibiotics were started for an acute pulmonary exacerbation (E). The third encounter occurred at the end of the antibiotic treatment course (T). The fourth encounter occurred at the next follow up visit in pulmonary clinic after completing IV antibiotic therapy, post-recovery (R). During each encounter, participants provided a respiratory sample (either BAL, expectorated sputum, or deep throat culture) for microbiome analysis. During the second encounter (E), the respiratory sample was collected the day IV antibiotic therapy was started. During the third encounter (T), the respiratory sample was collected before completion of antibiotic therapy. Serum samples were also collected for determination of antibiotic PK during the second and third encounters (E and T)^40^. Fuchs’ criteria were used to define APE during the second encounter (E)^41^. Study participants who experienced more than one APE requiring IV antibiotics during the study period were asked to participate for each treatment course (repeating the second through fourth encounters, E-R). Lung function testing was collected clinically following ATS spirometry guidelines^42^ and were reported using NHANES III reference values^43^.

### Data collection and pharmacokinetic (PK) analysis

REDCap electronic data capture tools were used to collect and store study data^44^. The following data was collected: age, gender, race/ethnicity, CFTR genotype, weight, height, medications, pulmonary function tests, and clinical culture data. A multiplex liquid chromatography mass spectrometry (LC-MS) assay in the PharmacoAnalytical laboratory (University of Southern California) was used to determine plasma drug concentrations of the beta-lactam antibiotics received. PK modeling of beta-lactam antibiotics to determine T > MIC was performed using Bayesian estimation in MW/Pharm (Version 3.80, Mediware)^45,46^.

### Pharmacodynamic (PD) analysis and determination of therapeutic versus subtherapeutic antibiotic exposure

The participants were stratified into therapeutic and subtherapeutic groups, on the basis of whether their beta-lactam exposure met appropriate T > MIC parameters (40% for carbapenems, 50% for penicillins, and 60% for cephalosporins)^22,23,37^. A full description of the determination of T > MIC has been published elsewhere, but in brief the highest MIC identified within a patient’s exacerbation culture against the beta-lactam antibiotics used for treatment was used^31^. If the patient grew only normal respiratory flora, the MIC used was the median MIC of the study patients who grew *P. aeruginosa*. MIC estimates from the literature were used for methicillin-sensitive *S. aureus* (MSSA). Participants on multiple beta-lactam antibiotics were deemed therapeutic if at least one beta-lactam antibiotic met the T > MIC parameters. Subjects who also grew methicillin-resistant *S. aureus* (MRSA) also had to receive directed anti-MRSA therapy to be considered therapeutic.

### Respiratory sample processing

Spontaneously expectorated sputum samples and oropharyngeal swabs were collected by clinical and research staff according to standardized procedures. BAL samples were only collected by clinical staff. Sputum and BAL samples were processed by washing first 1:1 v/v with sterile normal saline, mixing the sample 1:1 v/v with Sputasol (Fisher Healthcare, Houston TX), vortexing for 1 minute, and placing in a 37 °C heated water bath to homogenize the sample. The media of swab samples were transferred into a sterile microcentrifuge tube. All samples were then pelleted through centrifugation (12,000 *g* × 10 minutes). Supernatants were removed and pellets were frozen at −80 °C until they underwent DNA extraction.

### DNA extraction

DNA extraction was performed on the QIAsymphony SP (Qiagen, Valencia CA) using the DSP Virus/Pathogen Midi Kit and the Complex800_V6_DSP protocol. Prior to DNA extraction, pellets were thawed and a chemical lysis step was performed. Specifically, the bacterial pellet was suspended in 500 μl of combination enzyme solution, which included lysozyme (20 mg/mL) (Sigma-Aldrich, St. Louis MO) and lysostaphin (200 μg/mL) (Sigma-Aldrich, St. Louis MO) in nuclease free water. The pellet was then incubated at 37 °C for 30 minutes. The tube was then briefly centrifuged to remove drops from the inside of the lid before placing on the QIAsymphony.

### Next generation sequencing and bioinformatics

DNA (300 ng) was amplified for the V4 region (approximately 250 bp) of the 16S rRNA gene and libraries were sequenced on the MiSeq sequencing platform at the University of Michigan (Ann Arbor MI). Raw FASTQ files were processed in mothur (version 1.39.5)^47^. Default settings were used to minimize sequencing errors^48^. Clean sequences were aligned to the SILVA_v123 bacterial reference alignment at http://www.mothur.org. Sequences were clustered into OTUs at the 0.03 threshold. Samples were subsampled (rarefaction analysis) to the smallest sample size (2,239 sequences) to remove the effect of sample size bias on community composition. Alpha diversity was measured by the number of genera (No. genus; q = 0), abundance coverage estimator (Ace; q = 0), Chao (q = 0), Shannon Diversity Index (q = 1), and the Inverse Simpson’s Index (q = 2). No. genus, Ace, and Chao give more weight to richness. Shannon weights richness and evenness equally, while Simpson provides more weight to evenness^49^. Beta diversity between samples was measured using Morisita-Horn^49^. The OTU and taxonomy tables were also imported into Rstudio for subsequent analyses using *phyloseq* and *DESeq2* to determine differential abundance and create principle coordinates analysis (PCoA) plots^50,51^. A sensitivity analysis of sample type (lower respiratory, BAL or sputum, versus upper respiratory, OP swab) was performed to allow for elimination of samples whose diversity was potentially affected by the method of sample collection.

### Statistical analysis

Baseline participant characteristics between those with therapeutic and subtherapeutic beta-lactam antibiotic exposure were evaluated using chi-squared and t-test. We used Generalized Estimating Equations to compare measures that varied by exacerbation to account for the repeated exacerbations within patients, modeling an exchangeable correlation structure. For dichotomous outcomes, we assumed the binomial family and the log link function. For continuous outcomes, we assumed the Gaussian family and the identity link. We used robust variance estimators due to the small sample sizes.

We wanted to assess the impact of treatment on our primary outcomes across four measurement encounters: baseline (B), at the beginning of an exacerbation (E), at the end of treatment (T) and at the next post-recovery follow up visit (R)^12^. Since we did not expect a linear or even a quadratic change across encounters we chose to look at the change between any pair of encounters. When assessing significance, to account for the fact that all exacerbations are not independent (some patients have more than one exacerbation) we used the GLS random-effects model where exacerbation number acts as our indicator of time. We used robust variance estimates to account for the smaller sample size.

P values used to determine significance when using *DESeq2* to establish differential abundance were adjusted by the Benjamini-Hochberg adjustment^51^. PCoA plots using Bray and Jaccard distances with log transformed counts were created to visualize dissimilarities in microbial composition between therapeutic versus subtherapeutic antibiotic exposure and time of sample collection (B, E, T, or R). Permanova was used to evaluate differences in overall bacterial distributions. Significance was set at p < 0.05 and was determined using the *adonis* function of vegan in R^52^.

## Supplementary information

Supplemental Information

## Data Availability

The sequence dataset supporting the conclusions of this article is available in the NCBI SRA repository under BioProject PRJNA437613.
