# Supplementary material for: Changes in microbiome diversity following beta-lactam antibiotic treatment are associated with therapeutic versus subtherapeutic antibiotic exposure in cystic fibrosis
Source: Sci Rep. 2019 Feb 22;9:2534. doi: 10.1038/s41598-019-38984-y (PMC6385179; doi:10.1038/s41598-019-38984-y)
Supplement: Supplementary file 1 — Supplemental Information [file 41598_2019_38984_MOESM1_ESM.pdf]

Supplemental Information

Changes in microbiome diversity following beta-lactam antibiotic treatment are associated with therapeutic versus subtherapeutic antibiotic exposure in cystic fibrosis

Andrea Hahn MD, Hani Fanous MD, Caroline Jensen MS, Hollis Chaney MD, Iman Sami MD, Geovanny F. Perez MD, Anastassios C. Koumbourlis MD, MPH, Stan Louie PhD, James E. Bost PhD, John N. van den Anker MD, PhD, Robert J. Freishtat MD, MPH, Edith T. Zemanick MD, and Keith A. Crandall PhD

Supplemental Methods:

Sensitivity analysis for 16S rRNA sequencing results based on sample type

Respiratory samples [sputum, oropharyngeal (OP) swabs or bronchoalveolar lavage fluid (BAL)] were collected for 16S sequencing. Across all time points, 35% (n=18) of samples in the therapeutic group were sputum and 65% (n=33) were OP swabs. In the subtherapeutic group 79% (n=41) of the samples were sputum, 17% (n=9) were OP swabs, and 4% (n=2) were from BAL fluid. While the majority of patients had the same sample type obtained for all comparisons (80%), we examined the sequencing results of the 4 patients (AHCF08, ACHF09, ACHF11, and ACHF21) who had discordant sample types across comparisons (n=20 samples) using DESeq2. Three genera were differentially abundant with an adjusted p value < 0.05 in either sputum (Prevotellaceae_unclassified, log 2 fold change 4.71, p = 0.02) or OP swab samples (*Porphyromnas* and *Moraxella*, log 2 fold change -4.92 and -7.41, p = 0.002 and 0.006, respectively). We found that two samples were significantly affected, with a drop in the total sequences in the sample from 2239 to 342 (08c, OP swab) and 918 (08c2, sputum). The remaining 18 samples all maintained a total sequence count of ≥ 2086. We re-calculated alpha diversity measures pre- and post-removal of the three OTUs for the retained 18 samples and found a significant correlation (p < 0.001) in all measured alpha diversity measures: observed

genera R2 0.998, abundance coverage estimator (Ace) R2 0.981, Chao R2 0.959, Shannon R2 0.996, and Inverse Simpson R2 0.996. Thus, two samples (08c and 08c2) were removed from subsequent analysis based on sample collection type.

Supplemental Tables:

Supplemental Table 1. Changes in alpha diversity following antibiotic administration from Baseline

	Therapeutic* (N=14)[#]	Subtherapeutic (N=17)[‖]	P-value
No. genus[†] (mean, SD)			
Baseline to Treatment	-8.308 (12.932)	0.538 (8.628)	0.048
Baseline to Post-recovery	-1.286 (6.684)	2.700 (7.973)	0.095
Ace[†] (mean, SD)			
Baseline to Treatment	-8.170 (18.712)	1.286 (12.571)	<0.001
Baseline to Post-recovery	-3.977 (10.711)	5.449 (8.179)	0.008
Chao[†] (mean, SD)			
Baseline to Treatment	-7.095 (17.443)	1.443 (10.561)	<0.001
Baseline to Post-recovery	-2.410 (9.508)	5.257 (6.732)	0.009
Shannon Index[†] (mean, SD)			
Baseline to Treatment	-0.479 (0.833)	0.130 (0.881)	<0.001
Baseline to Post-recovery	-0.195 (0.510)	0.239 (0.343)	0.022
Inverse Simpson Index[†] (mean, SD)			
Baseline to Treatment	-2.404 (5.422)	0.103 (4.236)	<0.001
Baseline to Post-recovery	-1.716 (4.286)	1.051 (2.203)	<0.001

*Therapeutic and subtherapeutic categorization was based on each acute pulmonary exacerbation
[#]Between Baseline and Treatment, n=13, and between Baseline and Post-Recovery, n=14
[‖] Between Baseline and Treatment, n=13, and between Baseline and Post-Recovery, n=10
[†]No. genus (number of genera detected), Ace (abundance coverage estimator), and Chao are all measures of richness (q=0). Shannon Diversity Index equally weights richness and evenness (q=1). Inverse Simpson Index provides more weight to evenness (q=2).

Supplemental Table 2. Changes in alpha diversity following antibiotic administration from Exacerbation

	Therapeutic* (N=14)[#]	Subtherapeutic (N=17)[‖]	P-value
	Mean	Mean	
No. genus[†] (mean, SD)			
Exacerbation to Treatment	-7.615 (11.630)	-2.462 (11.759)	0.256
Exacerbation to Post-Recovery	-1.571 (13.860)	3.500 (13.109)	0.346
Ace[†] (mean, SD)			
Exacerbation to Treatment	-7.719 (14.509)	-5.663 (15.688)	0.260
Exacerbation to Post-Recovery	-4.710 (17.531)	3.186 (18.683)	0.302
Chao[†] (mean, SD)			
Exacerbation to Treatment	-7.936 (16.655)	-5.595 (16.689)	0.474
Exacerbation to Post-Recovery	-4.165 (18.045)	2.158 (17.908)	0.398
Shannon Index[†] (mean, SD)			
Exacerbation to Treatment	-0.324 (0.811)	-0.002 (1.080)	0.557
Exacerbation to Post-Recovery	-0.113 (0.692)	0.396 (0.987)	0.118
Inverse Simpson Index[†] (mean, SD)			
Exacerbation to Treatment	-1.767 (2.739)	0.171 (3.263)	0.148
Exacerbation to Post-Recovery	-1.245 (2.289)	1.586 (2.511)	0.003

*Therapeutic and subtherapeutic categorization was based on each acute pulmonary exacerbation
[#]Between Baseline and Treatment, n=13, and between Baseline and Post-Recovery, n=14
[‖] Between Baseline and Treatment, n=13, and between Baseline and Post-Recovery, n=10
[†]No. genus (number of genera detected), Ace (abundance coverage estimator), and Chao are all measures of richness (q=0). Shannon Diversity Index equally weights richness and evenness (q=1). Inverse Simpson Index provides more weight to evenness (q=2).

Supplemental Figures:

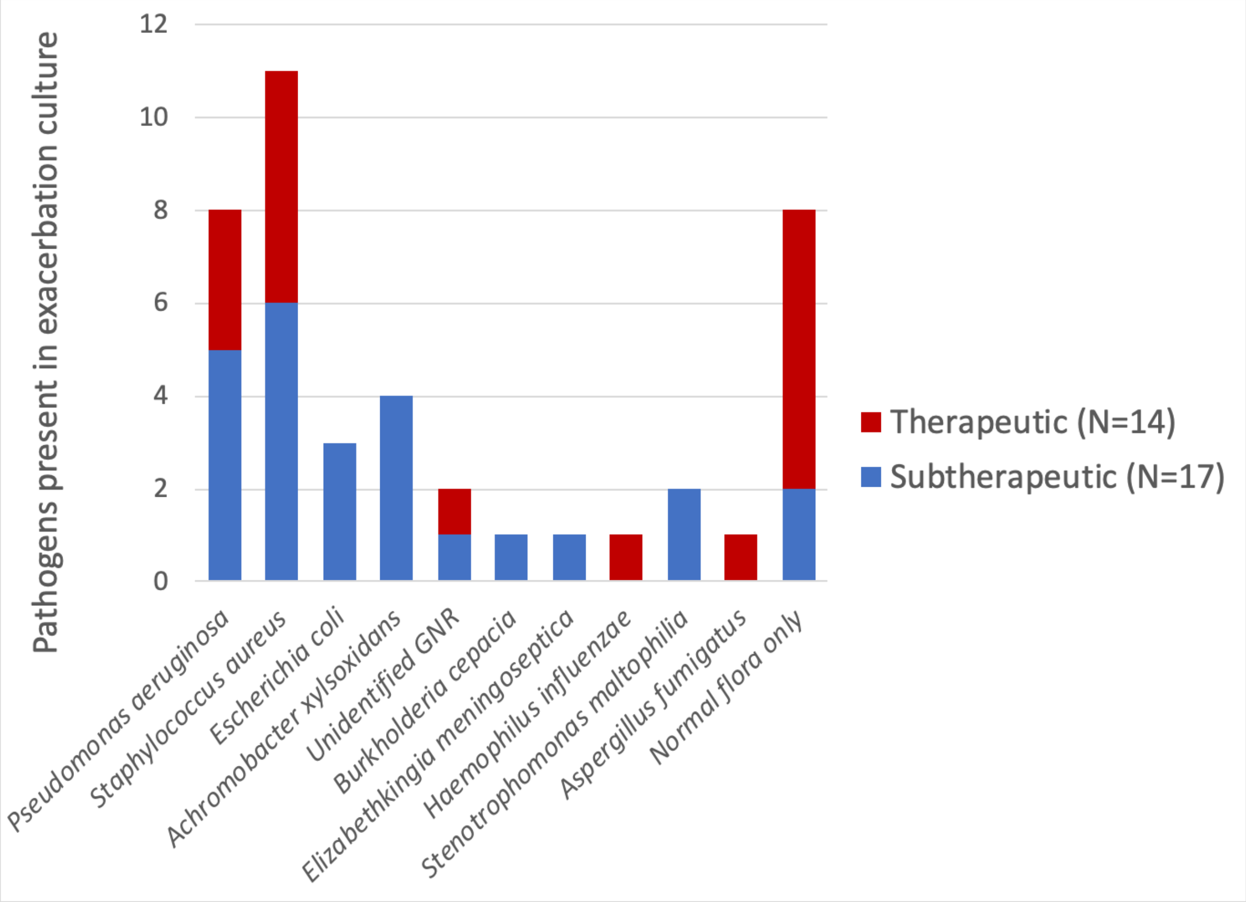

Supplemental Figure 1. Bacteria and Fungi Grown in Culture at Time of Pulmonary Exacerbation. Forty five percent of cultures (n=14) grew one pathogen, while 29% of cultures (n=9) grew more than one pathogen. Twenty six percent of cultures (n=8) grew only normal respiratory flora.

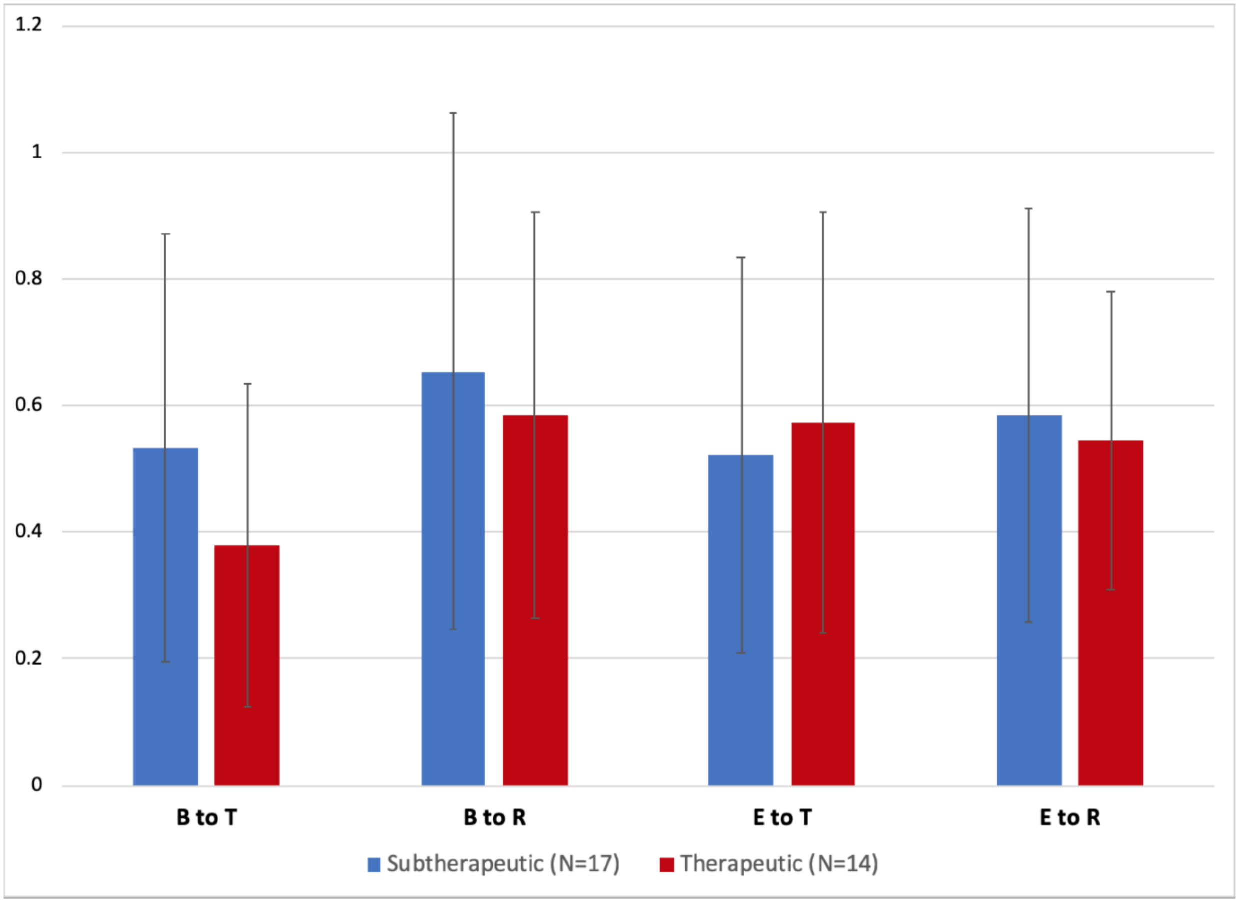

Supplemental Figure 2. Modeling the impact of therapeutic versus subtherapeutic treatments on airway beta diversity measures. Beta diversity was measured using Morisita-Horn (y axis). B, baseline; E, exacerbation; T, treatment; R, post-recovery. Error bars represent the standard deviation. The bar graph represents the mean Morisita-Horn value, and the error bars represent the standard deviation. There were no statistical differences noted between the two treatment types ($P<0.05$).

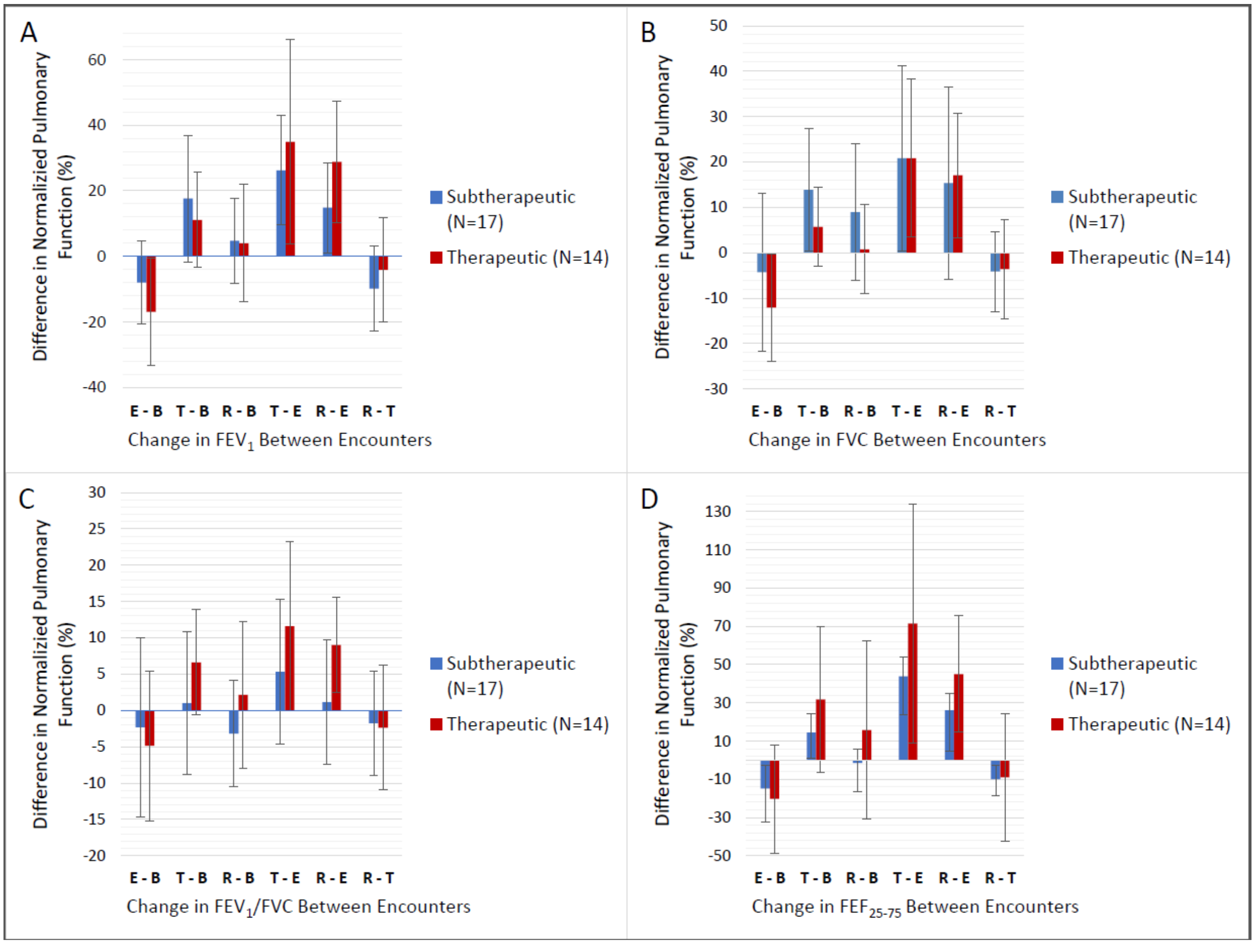

Supplemental Figure 3. Modeling the impact of therapeutic versus subtherapeutic treatments on pulmonary function measures for all exacerbations. The difference in pulmonary function between baseline, exacerbation, treatment, and post-recovery are labeled as E-B, T-B, etc. Normalized differences were calculated using the following equations: [(E-B)/B]*100, [(T-B)/B]*100, etc. Panel A. Forced expiratory volume in one second (FEV_1). Panel B. Forced vital capacity (FVC). Panel C. Ratio of forced expiratory volume in one second over forced vital capacity (FEV_1/FVC). Panel D. Forced expiratory flow at 25-75% (FEF_{25-75}). The bar graph represents the mean pulmonary function measurement, and the error bars represent the standard deviation. P-value was determined using generalized estimating equations. There were no statistical differences noted between the two treatment types ($P<0.05$).
